# Supplementary material for: Cystatin C as an adjunct to HbA1c may prove useful in predicting the development of diabetic complications in children and adolescents with type 1 diabetes
Source: J Diabetes Metab Disord. 2024 Apr 18;23(1):1251–7. doi: 10.1007/s40200-024-01419-1 (PMC11196488; doi:10.1007/s40200-024-01419-1)
Supplement: Supplementary file 1 — Supplementary Material 1 [file 40200_2024_1419_MOESM1_ESM.docx]

**Cystatin C as an adjunct to HbA1c may prove useful in predicting the development of diabetic complications in children and adolescents with type 1 diabetes**

Authors:

Charlotta Nilsson, MD, PhD^1^

Jonatan Dereke PhD^2^

^1^Department of Pediatrics, Department of Clinical Sciences, Helsingborg Hospital, Lund University, Helsingborg, Sweden Orchid 0002-0561-8526

^2^Department of Clinical Sciences, Diabetes Research Laboratory, Lund University, Lund, Sweden. Orchid 0000-0002-5653-8614

Corresponding author

Name: Charlotta Nilsson

Address: Helsingborgs Lasarett, Södra Vallgatan 5, S-251 87 Helsingborg, Sweden

Telephone number: 0046-4063024

Fax number: 0046-424063277

E-mail address: [charlotta.nilsson@med.lu.se](mailto:charlotta.nilsson@med.lu.se)

# Acknowledgement

We would like to acknowledge the nurses Anna Karlsson and Alexandra Larsson at Department of Paediatrics, Helsingborg Hospital for skilful help with collecting the blood samples as well as the rest of the diabetes team at the Department of Paediatrics, Helsingborg Hospital. Also, we would like to acknowledge Birgitte Ekholm at the Diabetes Research Laboratory for her excellent technical assistance with analysis of soluble proteins.

**Keywords:** type 1 diabetes, HbA1c, complications, proteins

**Abstract**

**Purpose**

# Complications from diabetes mellitus can occur over time and although glycosylated hemoglobin (HbA1c) is a good biomarker for glycaemic control, other factors also contribute to the development of complications in type 1 diabetes. More markers able to identify the risk of complications are needed. This study aimed to investigate plasma levels of FGF21, Cystatin C, lipocalin-2, and MMP-9 in children and adolescents with different duration of type 1 diabetes and possible correlation to HbA1c to identify potential biomarkers of future complication development.

**Methods**

Patients (n=244, 0-18 years) with type 1 diabetes, at Helsingborg’s Hospital, Sweden, were included in this study. Circulating levels of FGF21, Cystatin C, lipocalin-2, and MMP-9 were investigated in plasma using automated ELISA with the ELLA™ system and standardised controls.

**Results**

Cystatin C levels were elevated in patients with diabetes duration longer than 5 years (p<0.001). HbA1c and Cystatin C levels were inversely correlated for all participants (rs= −0.23, CI95: −0.35-−0.10; P<0.001). A stepwise multiple regression analysis showed that HbA1c (P<0.001) and Cystatin C (P=0.03) were associated to the duration of diabetes at sampling while MMP-9, lipocalin-2, and FGF21 did not reach statistical significance.

**Conclusion**

In conclusion, Cystatin C levels were higher in patients with diabetes duration longer than 5 years, and inverse correlation was found between HbA1c and Cystatin C levels as well as duration of diabetes. Cystatin C may prove useful as an adjunct to HbA1c in predicting eventual development of diabetic complications.

# Introduction

Complications from diabetes mellitus such as retinopathy, nephropathy, and neuropathy are related to hyperglycaemia, diabetes duration, age, blood pressure, and albuminuria [1, 2]. Glycosylated haemoglobin (HbA1c) is a good biomarker for glycaemic control since it measures average blood glucose level over the recent 2-3 months [3]. Different organizations for diabetes care have varying HbA1c goals but in Sweden, the target for optimal glycaemic control is HbA1c levels ≤48 mmol/mol for children and adolescents without the presence of severe hypoglycaemia or frequent mild hypoglycaemia [1, 4]. Besides HbA1c, continuous glucose monitoring (CGM) with Time in Range (TIR), defined as blood glucose between 3.9-10.0 mmol/L, and Time in Target (TIT) defined as blood glucose between 3.9-7.8 mmol/l are very useful as markers for glycaemic control [5]. Since type 1 diabetes often develops during childhood, children and adolescents affected by the disease sometimes already developed complications when reaching adulthood. A recently published study showed that to avoid retinopathy that needed laser or intraocular injections and microalbuminuria 32 years after diagnosis with type 1 diabetes, an HbA1c below 53 mmol/mol and as normal as possible should be recommended [6]. However, it seems that other factors are related to the development of complications in type 1 diabetes. More markers able to identify the risk of complications early, perhaps even before they occur, are needed.

Fibroblast growth factor 21 (FGF21) is a peptide hormone that regulates energy homeostasis and is increased in [ischemic heart disease](https://www.sciencedirect.com/topics/medicine-and-dentistry/ischemic-heart-disease), type 2 diabetes, insulin resistance, and dyslipidaemia where it has been shown to act as a compensatory reaction [7, 8]. Some studies have shown associations between FGF21 and nephropathy and retinopathy in type diabetes [7, 9] whereas another study of elderly patients with type 1 diabetes showed no such association [8].

Cystatin C is a non-glycosylated low-molecular-weight (13 kDa) protein that is found in nucleated cells without specificity for tissue and is independent of age and gender. Since it is filtered only by the glomerulus, it is considered a good marker for kidney function [10, 11]. It has potential as an early marker for diabetes related complications, since the association with a decrease in the glomerular filtration rate (GFR) and albumin to creatinine ratio (ACR) and progression to nephropathy in patients with diabetes have been shown [12, 13].

Lipocalin-2 is a novel adipokine which has been shown to relate to the level of inflammation in diseases and therefore linked to obesity and insulin resistance [14] Correlation between lipocalin-2 and vascular endothelial growth factor has been found in patients with proliferative diabetes retinopathy [15] and to retinopathy in patients with diabetes and overweight/obesity [16].

In zinc-dependent proteinase family Matrix metalloproteinase (MMP), MMP-9 is the largest molecule

and is involved in many inflammatory events [17, 18]. Higher levels of MMP-9 have been shown in patients with diabetes and increased with diabetes duration [19]. Inhibition of MMP-9 could have a role in the treatment of retinopathy [20].

Even though there are studies of markers connected to complications from diabetes, as mentioned above, they are primarily conducted on adults or young adults and not on children. Children that develop type 1 diabetes have had the disease for a long time when reaching adulthood, and more studies are needed on this population.

# Aim

# The first aim of this study was to investigate plasma levels of FGF21, Cystatin C, lipocalin 2 and MMP-9 in children and adolescents with different duration of type 1 diabetes. The second aim was to study possible correlation to HbA1c over time.

# Methods

## §Patients

In this study children and adolescents (up to the age of 18) diagnosed with type 1 diabetes at the Paediatric Department at Helsingborg Hospital in Sweden were included (n=244). Inclusion criteria was pre-existing type 1 diabetes or onset of type 1 diabetes during study time and exclusion criteria was age of 19 years or older. Information about the study were received both oral and written and for children up to 15 years of age their parents signed the written consent to participate in the study and from the age of 15 the adolescent, as well as their parents, signed the written consent. The participation rate was 100 % for those who were asked about the study at with clinical onset and diagnosis of type 1 diabetes. However, four were missed and not asked about the study. Among the children and adolescents at the Department of Paediatrics with pre-existing type 1 diabetes when the study started (n=166) two choose not to participate. For children and adolescents not at onset of type 1 diabetes, blood samples were collected once a year at the same time as their regular annual blood tests at the Department of Paediatrics at Helsingborg Hospital. After the age of 18 years adolescents in Sweden are transferred to the Department of Endocrinology and therefore did no longer participate at follow-up in this study. Blood was drawn into ethylenediaminetetraacetic acid (EDTA) plasma tubes and centrifuged at 2000 x g for 10 min at 20°C. Plasma was separated from blood cells and stored in -80°C until use.

At time of blood sample collection, the children´s and adolescent’s HbA1c age, weight and height were noted. The study was in accordance with the declaration of Helsinki and approved by the Regional Ethical Review Board in Lund, Sweden, Dnr 2006/599, 2013/693 and 2014/822.

## Laboratory methods

Soluble levels of Cystatin C, FGF21, lipocalin-2 and MMP-9 was analysed in patient plasma samples using the automated ELISA platform, ELLA™ (Bio-Techne ltd. Abingdon, UK). Samples were analysed in triplicates in Simple Plex Cartridge kits (Catalogue #SPCKB-PS-000401, SPCKB-PS-000509, SPCKB-PS-000661 and SPCKB-PS-007569) with human controls (Catalogue #898097, 898099, 898175 and 898185). Samples and controls were diluted 1:2000, 1:2, 1:200 and 1:100 for Cystatin C, FGF21, lipocalin-2 and MMP-9 respectively using Sample diluent SD13 (Catalogue #992517). Lower limit of quantification (LLOQ) was 2.69 pg/ml, 8.93 pg/ml, 17.2 pg/ml and 40 pg/ml for Cystatin C, FGF21, lipocalin-2 and MMP-9 respectively. IFCC-units mmol/mol were used for HbA1c and analyzed in Capillarys TERA Hemoglobin A1c Kits at diagnosis [21] and thereafter in Alere Afinion AS100 analyzer [22].

## Statistical analyses

Normality of continuous data was estimated using the D’Agostino-Pearson test prior to statistical analysis. Parametric data was presented as mean ± SD, while non-parametric data was presented as median followed by interquartile range. Continuous data was investigated using 2-way ANOVA or the Kruskal-Wallis H-test based on normality. Tukay-Kramer or Dunn’s post-hoc analysis applied for pairwise comparison of subgroups. For paired analyses, paired samples T-test or the Wilcoxon test for paired samples was used. Stepwise multiple regression was used to establish predictive strength of soluble proteins and diabetes duration. Associations in continuous variables was analysed with regression analysis or the Spearman-rank test depending on normality. The Chi-squared test was used to compare differences in sex between groups. P-values below 0.05 were considered statistically significant. Statistical analyses were performed using MedCalc Statistical Software version 19.6 (MedCalc Software Ltd, Ostend, Belgium; https://www.medcalc.org; 2020).

# Results

The diabetes duration for patients with type 1 diabetes included in this study was 0-16 years at blood sampling. In table 1 demographic information and biochemical data for the study participants are shown. Age and BMI was highest among those with diabetes duration >10 years (p<0.001), There was no significant difference between sex and diabetes duration. HbA1c levels were higher in patients with a diabetes duration <5 years (65.0 [50.5-88.5] mmol/mol) compared to patients with a diabetes duration of 5-10 years (55.0 [50.0-65.0] mmol/mol; P=0.005), but not in comparison to patients with a diabetes duration >10 years. Cystatin C levels were shown to be elevated in patients with a longer diabetes duration (696 [574-794] and 726 [616-795] ng/ml) for patients with a duration of 5-10 or >10 years respectively compared to patients with a diabetes duration <5 years (605 [522-708] ng/ml; p<0.001). Statistically significant differences in lipocalin-2 and MMP-9 could be observed when comparing patients with a diabetes duration <5 years and patients with a diabetes duration of 5-10 years (P=0.008 for both), but not in comparison to patients with a diabetes duration >10 years. FGF21 could be observed not to differ between any of the analysed groups.

HbA1c and Cystatin C levels were inversely correlated for all participants (r_s_= −0.23, CI^95^: −0.35 - −0.10; P<0.001). HbA1c was however positively correlated to MMP-9 (r_s_= 0.26, CI^95^: 0.13 - 0.36; P<0.001), lipocalin-2 (r_s_= 0.21, CI^95^: 0.07 - 0.34; P=0.002) and FGF-21 for all participants (r_s_= 0.26, CI^95^: 0.13 - 0.38; P<0.001)

(figure 1 panel A-D). A stepwise multiple regression analysis showed that HbA1c (P<0.001) and Cystatin C (P=0.03) were associated to the duration of diabetes at sampling while MMP-9, lipocalin-2 and FGF21 did not reach statistical significance (table 2).

In a sub-group of patients with type 1 diabetes blood was drawn at time of and after diagnosis, with a mean time between samplings 1.3 ± 0.5 years (n=42), the trajectory of circulating Cystatin C, MMP-9, lipocalin-2 and FGF21 was analysed. Cystatin C was shown to increase from diagnosis to follow-up (613 [527-707] ng/ml to 684 [603-743] ng/ml respectively; P=0.002). MMP-9 levels on the other hand decreased from diagnosis to follow-up (961 [682-1590] ng/ml to 689 [476-1210] ng/ml respectively; P=0.02). No statistically significant difference in lipocalin-2 or FGF21 could be observed from diagnosis to follow-up.

# Discussion

When investigating FGF21, Cystatin C, lipocalin-2, and MMP-9 in this study from diagnosis of type 1 diabetes over time, Cystatin C showed most promising results with increased values from diagnosis to follow-up and inverse correlation to HbA1c and duration of diabetes. Renal disease is one risk factor for premature mortality in diabetes [23]. Therefore, it is very important to find trustworthy markers that could lead to early detection of kidney damage and possibly earlier intervention. A previous study of 778 adults (aged 20 or older) investigating Cystatin C compared to creatinine based on eGFR showed that Cystatin C was found to reduce kidney function earlier, especially in patients with diabetes, indicating it could be used as an earlier marker of kidney damage [24]. When investigating children and young adults with type 1 diabetes (=779) with a median age of 16.2 years and median diabetes duration of 5.3 years, similar results were found. Of all patients, 30.2% had more severe kidney dysfunction when Cystatin C was used instead of creatinine for eGFR, and a linear correlation was found between Cystatin C and HbA1c levels [25]. Unfortunately, we have not measured creatinine in our study but there was still an increase in Cystatin C at our follow-up time (mean 1.3 years and higher levels after a longer diabetes duration. In Sweden, creatinine is analyzed every year in children and young adults with type 1 diabetes as part of a clinical control together with some other blood tests. Future inclusion of Cystatin C may prove beneficial in elucidating patients at increased risk of kidney damage.

Lipocalin-2 has been studied foremost in obese/overweight patients and type 2 diabetes showing association to retinopathy [15, 16] but significantly correlation with a decreased glomerular filtration rate has been shown in young adults with typ1 1 diabetes [26]. MMP-9, as a marker of tissue damage, has been shown to increase in patients with longer diabetes duration [19]. In a study of adult patients with type 1 diabetes without vascular complications (n=47) MMP-9 was higher than in controls without diabetes and higher in patients with retinopathy than patients without retinopathy [27]. It is difficult to draw any conclusions from previous studies compared to our results since lipocalin-2 and MMP-9 levels only significantly changed when comparing patients with a diabetes duration <5 years.

In a recent study where optical coherence tomography angiography (OCTA) was used, higher levels of FGF21 were found in children and adolescents with type 1 diabetes (n=100) compared to the same number of age, gender, and Tanner-matched healthy controls (n=100). FGF21 was also higher among patients with type 1 diabetes and OCTA changes compared to those without [28]. We could not in our study however, observe any difference in FGF21 levels with regard to diabetes duration. In adults with type 1 diabetes, circulating FGF21 levels were lower compared to healthy controls, and no association with diabetic complications was found [29].The importance of glycemic control and that hyperglycemia and high HbA1c levels decrease the time to development of diabetic complications is well known [2]. Findings of other clear and reliable markers for early detection of complications could help health carers with early intervention and maybe lead to more personalized and improved individual treatments. As previously mentioned, studies have shown an association with circulating proteins for vascular disease in both children and adults with type 1 diabetes [26-29]. In our own previous study we found sCD163 levels increased in patients with recent-onset type 1 diabetes and the levels increased with higher HbA1c [30].

With this study we aimed to find potential markers of future diabetic complications that could easily be measured in a small volume blood sample. Since the patients included in this study are still young and have not developed diabetes related complications, future studies following these patients are warranted to properly establish if the analysed proteins could have potential as biomarkers.

Strengths of this study are the high sensitivity and precision of the commercially available assays used to analyse blood samples and that all children and adolescents with type 1 diabetes from our geographical area at this time were included in the study. Limitations were that some patients had a short follow-up time and not all patients were followed from diagnosis of type 1 diabetes.

In conclusion, Cystatin C levels were higher in patients with diabetes duration longer than 5 years, and inverse correlation was found between HbA1c and Cystatin C levels as well as duration of diabetes. Cystatin C may prove useful as an adjunct to HbA1c in predicting eventual development of diabetic complications.

## Statements and Declarations

On behalf of all authors, the corresponding author states that there is no conflict of interest.

## Data availability statement

The data that support the findings of this study are available on request from the corresponding author, [CN]. The data are not publicly available due to [restrictions containing information that could compromise the privacy of research participants].

# References

1. Diabetes Control and Complications Trial Research Group, Nathan DM, Genuth S, Lachin J, Cleary P, Crofford O, Davis M, Rand L, Siebert C. The effect of intensive treatment of diabetes on the development and progression of long-term complications in insulin-dependent diabetes mellitus. N Engl J Med. 1993;329:977-86. doi: 10.1056/NEJM199309303291401
2. American Diabetes Association Professional Practice Committee; 2. Classification and Diagnosis of Diabetes: Standards of Medical Care in Diabetes—2022. Diabetes Care 2022;45(Suppl 1):17–38. [doi.: 10.2337/dc22-S002](https://doi.org/10.2337/dc22-S002)
3. Bunn HF, Gabbay KH, Gallop PM. The glycosylation of hemoglobin: relevance to diabetes mellitus. Science 1978;200:21–27. doi: 10.1126/science.635569
4. DiMeglio LA, Acerini CL, Codner E, et al. ISPAD Clinical Practice Consensus Guidelines 2018: glycemic control targets and glucose monitoring for children, adolescents, and young adults with diabetes. Pediatr Diabetes 2018;19(Suppl. 27):105–114. doi: 10.1111/pedi.12737
5. Battelino T, Danne, T, Bergenstal RM et al. Clinical targets for continuous glucose monitoring data interpretation: Recommendations from the international consensus on Time in Range. Diabetes Care 2019;42:1593-03. doi: 10.2337/dci19-0028
6. Arnqvist HJ, Westerlund MC, Fredrikson M, Ludvigsson J, Nordwall M. Impact of HbA1c Followed 32 Years From Diagnosis of Type 1 Diabetes on Development of Severe Retinopathy and Nephropathy: The VISS Study. Diabetes Care. 2022;45:2675-82. doi: 10.2337/dc22-0239
7. Heidari Z, Hasanpour M. The serum fibroblast growth factor 21 is correlated with retinopathy in patients with type 2 diabetes mellitus. Diabetes Metab Syndr. 2021;15:102296. doi: 10.1016/j.dsx.2021.102296
8. Taniguchi H, Nirengi S, Ishihara K, Sakane N. Association of serum fibroblast growth factor 21 with diabetic complications and insulin dose in patients with type 1 diabetes mellitus. PLoS One. 2022 22;17:e0263774. doi:10.1371/journal.pone.0263774
9. Jian WX, Peng WH, Jin J et al. Association between serum fibroblast growth factor 21 and diabetic nephropathy. Metabolism. 2012;61:853-9. doi: 10.1016/j.metabol.2011.10.012
10. Roos JF, Doust J, Tett SE, Kirkpatrick CMJ, Diagnostic accuracy of cystatin C compared to serum creatinine for the estimation of renal dysfunction in adults and children—a meta-analysis, Clin Biochem. 2007;40:383-91. doi: 10.1016/j.clinbiochem.2006.10.026
11. Dharnidharka VR, Kwon C, Stevens G, Serum cystatin C is superior to serum creatinine as a marker of kidney function: a meta-analysis. Am J Kidney Dis. 2002;40:221-6. doi: 10.1053/ajkd.2002.34487
12. Kim S.S., Song S.H., Kim I.J et al. Urinary cystatin C and tubular proteinuria predict progression of diabetic nephropathy. Diabetes Care. 2013;36:656–61. doi: 10.2337/dc12-0849
13. Jeon Y.L., Kim M.H., Lee W.I., Kang S.Y. Cystatin C as an early marker of diabetic nephropathy in patients with type 2 diabetes. Clin. Lab. 2013;59:1221–29. doi: 10.7754/Clin.Lab.2013.120804
14. Li D, Yan Sun W, Fu B, et al. Lipocalin-2-the myth of its expression and function. Basic Clin Pharmacol Toxicol. 2020;127:142-51 doi: 10.1111/bcpt.13332
15. Wang H, Lou H, Li Y et al. Elevated vitreous Lipocalin-2 levels of patients with proliferative diabetic retinopathy. BMC Ophthalmol. 2020;20:260. doi: 10.1186/s12886-020-01462-5
16. Zhang, Y., Song, X., Qi, T. et al. Association between lipocalin-2 levels and diabetic retinopathy in patients with overweight/obese type 2 diabetes mellitus.  Ir J Med Sci. 2023. doi: 10.1007/s11845-023-03365-y. Epub ahead of print
17. Itoh Y, Seiki M. CMT1-MMP: a potent modifier of pericellular microenvironment. J Cell Physiol. 2006;206:1–8. doi: 10.1002/jcp.20431
18. Dufour A, Sampson NS, Zucker S, Cao J. Role of the hemopexin domain of matrix metalloproteinases in cell migration. J Cell Physiol. 2008;217:643–51. doi: 10.1002/jcp.21535
19. Ünal A, Baykal O, Öztürk N. Comparison of matrix metalloproteinase 9 and 14 levels in vitreous samples in diabetic and non-diabetic patients: a case control study. Int J Retina Vitreous. 2022;8:44. doi: 10.1186/s40942-022-00394-0
20. Solanki A, Bhatt LK, Johnston TP, Prabhavalkar KS. Targeting matrix metalloproteinases for diabetic retinopathy: the way ahead? Curr Protein Pept Sci. 2019;20:324–33. doi: 10.2174/1389203719666180914093109
21. Jaisson S, Leroy N, Meurice J, Guillard E, Gillery P. First evaluation of Capillarys 2 Flex Piercing® (Sebia) as a new analyzer for HbA1c assay by capillary electrophoresis. Clin Chem Lab Med. 2012;50:1769–75. doi: 10.1515/cclm-2012-0017
22. Nathan DM, Griffin A, Perez FM, Basque E, Do L, Steiner B. Accuracy of a Point-of-Care Hemoglobin A1c Assay. J Diabetes Sci Technol. 2019;**13**:1149–53. doi: 10.1177/1932296819836101
23. He Z. Diagnosis and Treatment of Diabetic Nephropathy in Type 1 and Type 2 Diabetes Patients. J. Mol. Biomark. Diagn. 2016;7:5. doi: 10.4172/2155-9929.1000295
24. Tsai CW, Grams ME, Inker LA, Coresh J, Selvin E. Cystatin C- and creatinine-based estimated glomerular filtration rate, vascular disease, and mortality in persons with diabetes in the U.S. Diabetes Care. 2014;37:1002-8. doi: 10.2337/dc13-1910
25. Stankute I, Radzeviciene L, Monstaviciene A, Dobrovolskiene R, Danyte E, Verkauskiene R. Serum Cystatin C as a Biomarker for Early Diabetic Kidney Disease and Dyslipidemia in Young Type 1 Diabetes Patients. Medicina (Kaunas). 2022;;58:218. doi: 10.3390/medicina58020218
26. Lee JH, Yang FJ, Tsai WY et al. Serum neutrophil gelatinase-associated lipocalin as a potential biomarker of diabetic kidney disease in patients with childhood-onset type 1 diabetes. J Formos Med Assoc. 2022;121:832-40. doi: 10.1016/j.jfma.2021.06.022.
27. Jacqueminet S, Ben Abdesselam O, Chapman MJ et al. Elevated circulating levels of matrix metalloproteinase-9 in type 1 diabetic patients with and without retinopathy. Clin Chim Acta. 2006;367:103-7. doi: 10.1016/j.cca.2005.11.029
28. Sherif EM, Matter RM, Salah NY, Abozeid NEH, Atif HM, Tantawy NM. Changes in early optical coherence tomography angiography among children and adolescents with type 1 diabetes: Relation to fibroblast growth factor 21. Diabetes Metab Res Rev. 2023;39:e3598. doi: 10.1002/dmrr.3598
29. Taniguchi H, Nirengi S, Ishihara K, Sakane N. Association of serum fibroblast growth factor 21 with diabetic complications and insulin dose in patients with type 1 diabetes mellitus. PLoS One. 2022;17:e0263774. doi: 10.1371/journal.pone.0263774
30. Dereke J, Nilsson C. HbA1c levels and circulating inflammatory proteins at onset of type 1 diabetes in children and adolescents. J Diabetes Metab Disord. 2022;21:1427-32. doi: 10.1007/s40200-022-01075-3

.

Table 1: Demographic information and biochemical data for the study participants. ANOVA or Kruskal-Wallis H-test was used to compare variables between groups. Chi-square was used to investigate differences in sex.

|  | <5 years | 5-10 years | >10 years | P-value |
| --- | --- | --- | --- | --- |
| N | 137 | 73 | 34 | N/A |
| Age (years) | 11.0 ± 4.5 | 13.5 ± 2.8 | 15.2 ± 2.3 | <0.001 |
| BMI (kg/m^2^) | 19.1 ± 4.6 | 21.0 ± 4.3 | 22.4 ± 4.2 | <0.001 |
| Sex (m/f) | 73/64 | 46/27 | 18/16 | 0.37 |
| Diabetes duration (years) | 0.0 [0.0-3.0] | 7.0 [6.0-8.0] | 11.0 [10.0-13.0] | N/A |
| HbA1c (mmol/mol) | 65.0 [50.5-88.5] | 55.0 [50.0-65.0] | 61.0 [55.5-67.0] | 0.005* |
| Cystatin C (ng/ml) | 605 [522-708] | 696 [574-794] | 726 [616-795] | <0.001 |
| Lipocalin-2 (ng/ml) | 479 [313-796] | 351 [259-550] | 429 [266-780] | 0.008* |
| MMP-9 (ng/ml) | 1003 [722-1530] | 823 [502-1144] | 1076 [760-1539] | 0.008* |
| FGF21 (pg/ml) | 45.7 [27.6-81.5] | 44.9 [29.3-85.9] | 54.5 [35.6-86.8] | NS |

*: Post hoc pairwise analysis of groups using Dunn’s test showed differences between the <5 and 5-10 years’ groups, but not for the >10 years’ group.

Table 2: Regression equation for the stepwise multiple regression analysis with diabetes duration as dependable variable. HbA1c and Cystatin C remained significantly associated while lipocalin-2, MMP-9 and FGF-21 did not reach statistical significance.

| Independent variable | Coefficient | Std error | t | P | r_partial_ | r_semipartial_ |
| --- | --- | --- | --- | --- | --- | --- |
| (Constant) | 6.2876 |  |  |  |  |  |
| HbA1c | -0.07001 | 0.01917 | -3.652 | <0.001 | -0,2912 | 0,2841 |
| Cystatin C | 0.004734 | 0.002220 | 2.132 | 0.03 | 0.1749 | 0,1658 |
| Lipocalin-2* |  |  |  | NS |  |  |
| MMP-9* |  |  |  | NS |  |  |
| FGF-21* |  |  |  | NS |  |  |
| F-ratio | 10.6501 |  |  |  |  |  |
| Significance level | <0.0001 |  |  |  |  |  |

* Variables which did not reach significance and was removed from the model

#
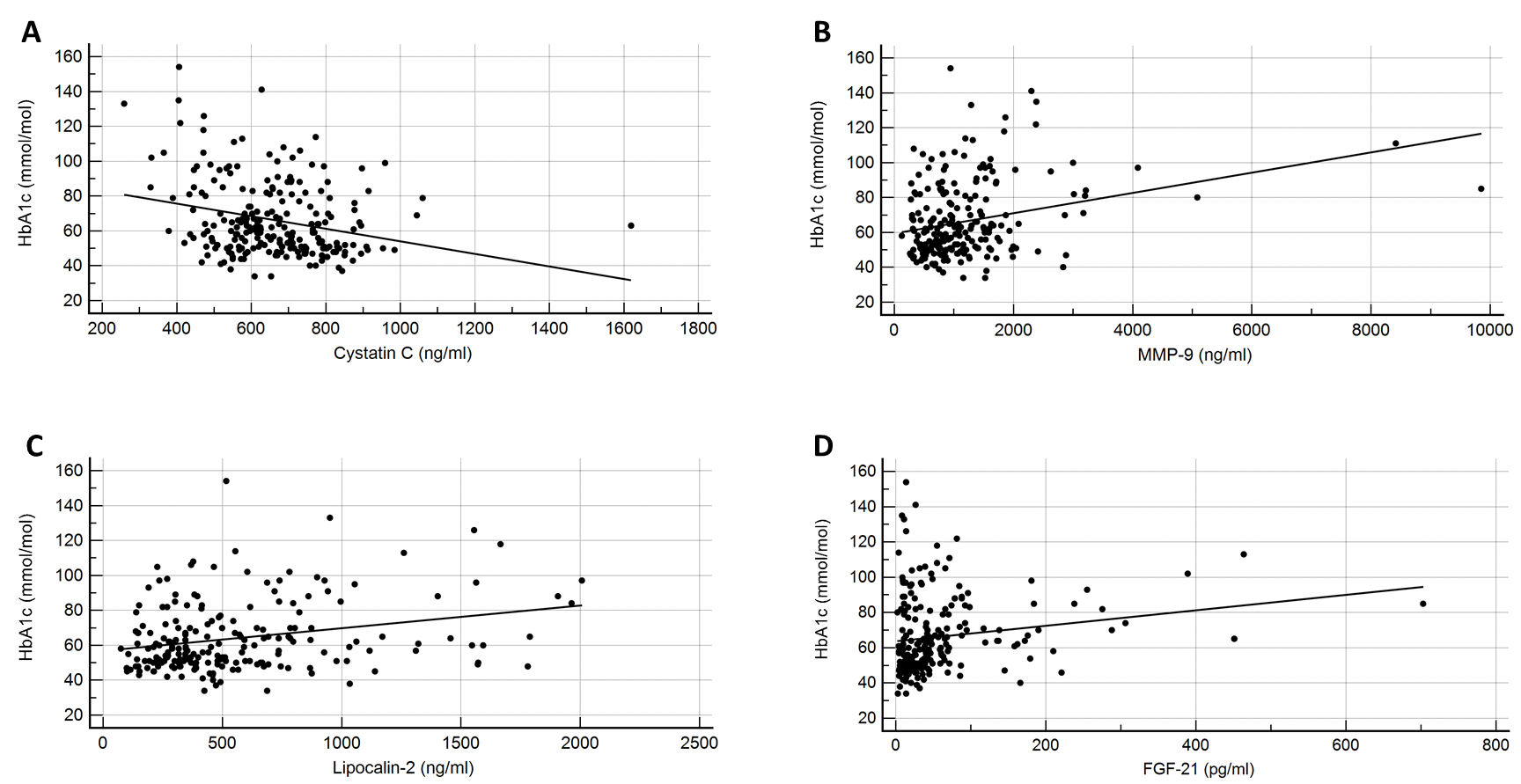


Figure 1: Correlations between HbA1c and soluble proteins investigated with the Spearman rank test. A: Inverse correlation to Cystatin C (r_s_= −0.23, CI^95^: −0.35 - −0.10; P<0.001). B-D: Positive correlations to MMP-9 (r_s_= 0.26, CI^95^: 0.13 - 0.36; P<0.001), lipocalin-2 (r_s_= 0.21, CI^95^: 0.07 - 0.34; P=0.002) and FGF-21 (r_s_= 0.26, CI^95^: 0.13 - 0.38; P<0.001).
