# Supplementary material for: Cystatin C as an adjunct to HbA1c may prove useful in predicting the development of diabetic complications in children and adolescents with type 1 diabetes
Source: J Diabetes Metab Disord. 2024 Apr 18;23(1):1251–7. doi: 10.1007/s40200-024-01419-1 (PMC11196488; doi:10.1007/s40200-024-01419-1)
Supplement: Supplementary file 2 — Supplementary Material 2 [file 40200_2024_1419_MOESM2_ESM.docx]

Dear Editor-in-chief

We would appreciate if you considered the enclosed manuscript entitled “Title: **Cystatin C as an adjunct to HbA1c may prove useful in predicting the development of diabetic complications in children and adolescents with type 1 diabetes***”* for submission. We have tried to improve the manuscript according to the reviewers comments.

In this study we wanted to investigate plasma levels of FGF21, Cystatin C, Lipocalin-2, and MMP-9 at diagnosis of type 1 diabetes in children and adolescents and follow the levels of these soluble proteins with diabetes progression as well as studying their correlation to HbA1c.

Kind regards

Charlotta Nilsson

MD, PhD

Department of Pediatrics

Department of Clinical Science

Helsingborg Hospital

Lund University
S- 251 87 Helsingborg

Sweden

E-mail: [Charlotta.Nilsson@med.lu.se](mailto:Charlotta.Nilsson@med.lu.se)
